# {μ-6,6′-Dimeth­oxy-2,2′-[propane-1,3-diylbis(nitrilo­methyl­idyne)]­diphenolato}­trinitratocopper(II)lutetium(III) acetone solvate

**DOI:** 10.1107/S1600536809009581

**Published:** 2009-03-25

**Authors:** Jing-Chun Xing, Yong-Mei Xu, Xiao-Guang Cui, Wen-Zhi Li

**Affiliations:** a Department of Anaesthesiology, The Second Affiliated Hospital, Harbin Medical University, Harbin 150081, People’s Republic of China

## Abstract

In the title complex, [CuLu(C_19_H_20_N_2_O_4_)(NO_3_)_3_]·CH_3_COCH_3_, the Cu^II^ ion is four-coordinated in a square-planar geometry by two O atoms and two N atoms from the deprotonated Schiff base. The Lu^III^ ion is ten-coordinate, chelated by three nitrate groups and linked to the four O atoms of the deprotonated Schiff base. A mol­ecule of acetone is present as a solvate.

## Related literature

For copper–lanthanide complexes of the same Schiff base, see: Elmali & Elerman (2003 ▶, 2004 ▶).
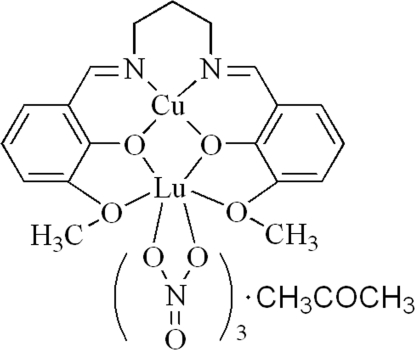


## Experimental

### 

#### Crystal data

[CuLu(C_19_H_20_N_2_O_4_)(NO_3_)_3_]·C_3_H_6_O
*M* *_r_* = 822.99
Triclinic, 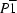

*a* = 9.4070 (19) Å
*b* = 12.135 (2) Å
*c* = 13.510 (3) Å
α = 73.03 (3)°
β = 87.04 (3)°
γ = 72.32 (3)°
*V* = 1404.2 (6) Å^3^
*Z* = 2
Mo *K*α radiation
μ = 4.33 mm^−1^
*T* = 295 K
0.34 × 0.28 × 0.20 mm

#### Data collection

Rigaku R-AXIS RAPID diffractometer
Absorption correction: multi-scan (*ABSCOR*; Higashi, 1995 ▶) *T*_min_ = 0.220, *T*_max_ = 0.420
13735 measured reflections
6345 independent reflections
5817 reflections with *I* > 2σ(*I*)
*R*_int_ = 0.063

#### Refinement

*R*[*F* ^2^ > 2σ(*F* ^2^)] = 0.056
*wR*(*F* ^2^) = 0.149
*S* = 1.05
6345 reflections
392 parameters
H-atom parameters constrained
Δρ_max_ = 4.51 e Å^−3^
Δρ_min_ = −2.38 e Å^−3^

### 

Data collection: *RAPID-AUTO* (Rigaku, 1998 ▶); cell refinement: *RAPID-AUTO*; data reduction: *CrystalStructure* (Rigaku/MSC, 2002 ▶); program(s) used to solve structure: *SHELXS97* (Sheldrick, 2008 ▶); program(s) used to refine structure: *SHELXL97* (Sheldrick, 2008 ▶); molecular graphics: *ORTEPII* (Johnson, 1976 ▶); software used to prepare material for publication: *SHELXL97*.

## Supplementary Material

Crystal structure: contains datablocks I, global. DOI: 10.1107/S1600536809009581/fj2197sup1.cif

Structure factors: contains datablocks I. DOI: 10.1107/S1600536809009581/fj2197Isup2.hkl

Additional supplementary materials:  crystallographic information; 3D view; checkCIF report

## Figures and Tables

**Fig. 1.**
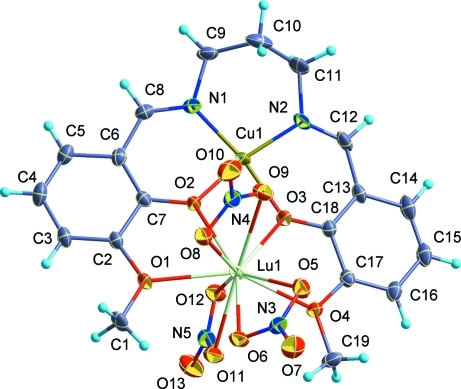
The molecular structure of (I), showing 40% probability displacement ellipsoids. The acetone molecule of solvation has been omitted for clarity.

## supplementary crystallographic information

### Comment

As shown in Fig. 1, the octodentate Schiff base ligand links Cu and Lu atoms into a dinuclear complex through two phenolate O atoms, which is similar with the bonding reported for another copper-lanthanide complex of the same ligand (Elmali & Elerman, 2003, 2004). The Lu^III^ centre in (I) is ten-coordinated by four oxygen atoms from the ligand and six oxygen atoms from three nitrate ions. The Cu^II^ center is four-coordinate by two nitrogen atoms and two oxygen atoms from the ligand. And one molecular acetone is dissociative in the complex.

### Experimental

The title complex was obtained by the treatment of copper(II) acetate monohydrate(0.0499 g, 0.25 mmol) with the Schiff base(0.0855 g, 0.25 mmol) in methanol/acetone (20 ml:5 ml) at room temperature. Then the mixture was refluxed for 3 h after the addition of lutetium (III) nitrate hexahydrate (0.1172 g, 0.25 mmol). The reaction mixture was cooled and filtered; diethyl ether was allowed to diffuse slowly into the solution of the filtrate. Single crystals were obtained after several days. Analysis calculated for C_22_H_26_CuN_5_O_14_Lu: C, 32.08; H, 3.16; Cu, 7.72; N, 8.51; Lu, 21.26; found: C, 32.11; H, 3.10; Cu, 7.76; N, 8.48; Lu, 21.22%.

### Refinement

H atoms bound to C atoms were placed in calculated positions and treated as riding on their parent atoms, with C—H = 0.93 Å (aromatic C), C—H = 0.97 Å (methylene C), C—H = 0.98 Å (methine C), and with *U*_iso_(H) = 1.2Ueq(C) or C—H = 0.96 Å (methyl C) and with *U*_iso_(H) = 1.5Ueq(C).

### Crystal data

**Table:**

[CuLu(C_19_H_20_N_2_O_4_)(NO_3_)_3_]·C_3_H_6_O	*Z* = 2
*M**_r_* = 822.99	*F*(000) = 810
Triclinic, *P*1	*D*_x_ = 1.946 Mg m^−^^3^
Hall symbol: -p 1	Mo *K*α radiation, λ = 0.71073 Å
*a* = 9.4070 (19) Å	Cell parameters from 12159 reflections
*b* = 12.135 (2) Å	θ = 3.2–27.4°
*c* = 13.510 (3) Å	µ = 4.33 mm^−^^1^
α = 73.03 (3)°	*T* = 295 K
β = 87.04 (3)°	Prism, green
γ = 72.32 (3)°	0.34 × 0.28 × 0.20 mm
*V* = 1404.2 (6) Å^3^	

### Data collection

**Table:**

Rigaku R-AXIS RAPID diffractometer	6345 independent reflections
Radiation source: fine-focus sealed tube	5817 reflections with *I* > 2σ(*I*)
graphite	*R*_int_ = 0.063
Detector resolution: 10.000 pixels mm^-1^	θ_max_ = 27.4°, θ_min_ = 3.2°
ω scans	*h* = −11→12
Absorption correction: multi-scan (*ABSCOR*; Higashi, 1995)	*k* = −15→15
*T*_min_ = 0.220, *T*_max_ = 0.420	*l* = −17→17
13735 measured reflections	

### Refinement

**Table:**

Refinement on *F*^2^	Primary atom site location: structure-invariant direct methods
Least-squares matrix: full	Secondary atom site location: difference Fourier map
*R*[*F*^2^ > 2σ(*F*^2^)] = 0.056	Hydrogen site location: inferred from neighbouring sites
*wR*(*F*^2^) = 0.149	H-atom parameters constrained
*S* = 1.05	*w* = 1/[σ^2^(*F*_o_^2^) + (0.1022*P*)^2^] where *P* = (*F*_o_^2^ + 2*F*_c_^2^)/3
6345 reflections	(Δ/σ)_max_ = 0.001
392 parameters	Δρ_max_ = 4.51 e Å^−^^3^
0 restraints	Δρ_min_ = −2.38 e Å^−^^3^

### Special details

**Table:**

Geometry. All e.s.d.'s (except the e.s.d. in the dihedral angle between two l.s. planes) are estimated using the full covariance matrix. The cell e.s.d.'s are taken into account individually in the estimation of e.s.d.'s in distances, angles and torsion angles; correlations between e.s.d.'s in cell parameters are only used when they are defined by crystal symmetry. An approximate (isotropic) treatment of cell e.s.d.'s is used for estimating e.s.d.'s involving l.s. planes.
Refinement. Refinement of *F*^2^ against ALL reflections. The weighted *R*-factor *wR* and goodness of fit *S* are based on *F*^2^, conventional *R*-factors *R* are based on *F*, with *F* set to zero for negative *F*^2^. The threshold expression of *F*^2^ > σ(*F*^2^) is used only for calculating *R*-factors(gt) *etc*. and is not relevant to the choice of reflections for refinement. *R*-factors based on *F*^2^ are statistically about twice as large as those based on *F*, and *R*- factors based on ALL data will be even larger.

### Fractional atomic coordinates and isotropic or equivalent isotropic displacement parameters (Å^2^)

**Table:**

	*x*	*y*	*z*	*U*_iso_*/*U*_eq_	
Lu1	0.284988 (19)	0.378525 (16)	0.234889 (13)	0.03573 (12)	
Cu1	0.24429 (6)	0.25725 (5)	0.49539 (4)	0.03148 (15)	
O1	0.0395 (4)	0.5365 (3)	0.1965 (3)	0.0382 (7)	
O2	0.1287 (3)	0.3741 (3)	0.3751 (3)	0.0353 (7)	
O3	0.4010 (4)	0.2710 (3)	0.3973 (3)	0.0403 (8)	
O4	0.5591 (4)	0.3498 (3)	0.2448 (3)	0.0399 (8)	
O5	0.4248 (4)	0.2376 (4)	0.1369 (3)	0.0477 (9)	
O6	0.3037 (4)	0.4173 (3)	0.0479 (3)	0.0435 (8)	
O7	0.4124 (6)	0.2842 (5)	−0.0318 (4)	0.0645 (12)	
O8	0.0996 (4)	0.2996 (3)	0.1767 (3)	0.0481 (9)	
O9	0.2459 (6)	0.1682 (4)	0.3012 (4)	0.0527 (10)	
O10	0.0812 (6)	0.1186 (4)	0.2316 (5)	0.0691 (13)	
O11	0.3233 (5)	0.5776 (4)	0.1485 (3)	0.0468 (9)	
O12	0.3121 (5)	0.5301 (4)	0.3144 (3)	0.0473 (9)	
O13	0.3323 (6)	0.7056 (4)	0.2309 (4)	0.0687 (13)	
O14	0.2009 (10)	0.0886 (5)	−0.0752 (5)	0.103 (2)	
N1	0.0629 (5)	0.2641 (4)	0.5757 (3)	0.0351 (8)	
N2	0.3899 (5)	0.1300 (4)	0.6000 (3)	0.0434 (10)	
N3	0.3804 (5)	0.3120 (4)	0.0479 (3)	0.0405 (9)	
N4	0.1412 (5)	0.1922 (4)	0.2357 (4)	0.0467 (10)	
N5	0.3232 (5)	0.6071 (4)	0.2308 (4)	0.0446 (10)	
C1	−0.0128 (7)	0.6110 (5)	0.0917 (4)	0.0504 (14)	
H1A	−0.0932	0.5887	0.0704	0.076*	
H1B	0.0677	0.5989	0.0454	0.076*	
H1C	−0.0472	0.6945	0.0900	0.076*	
C2	−0.0664 (5)	0.5331 (4)	0.2710 (4)	0.0317 (9)	
C3	−0.2132 (5)	0.6058 (4)	0.2582 (4)	0.0392 (11)	
H3	−0.2483	0.6626	0.1948	0.047*	
C4	−0.3080 (6)	0.5938 (5)	0.3400 (5)	0.0475 (13)	
H4	−0.4068	0.6428	0.3307	0.057*	
C5	−0.2588 (5)	0.5114 (5)	0.4338 (5)	0.0431 (11)	
H5	−0.3230	0.5053	0.4884	0.052*	
C6	−0.1076 (5)	0.4343 (4)	0.4478 (4)	0.0329 (9)	
C7	−0.0127 (5)	0.4448 (4)	0.3680 (4)	0.0306 (9)	
C8	−0.0661 (6)	0.3413 (4)	0.5460 (4)	0.0366 (10)	
H8	−0.1414	0.3370	0.5930	0.044*	
C9	0.0686 (7)	0.1741 (6)	0.6806 (4)	0.0483 (13)	
H9A	0.0916	0.2063	0.7335	0.058*	
H9B	−0.0286	0.1618	0.6938	0.058*	
C10	0.1835 (9)	0.0554 (5)	0.6873 (5)	0.0611 (18)	
H10A	0.1643	−0.0060	0.7461	0.073*	
H10B	0.1747	0.0327	0.6253	0.073*	
C11	0.3383 (9)	0.0592 (7)	0.6987 (5)	0.074 (2)	
H11A	0.4056	−0.0228	0.7192	0.089*	
H11B	0.3427	0.0954	0.7532	0.089*	
C12	0.5322 (6)	0.1036 (5)	0.5942 (4)	0.0448 (12)	
H12	0.5884	0.0450	0.6507	0.054*	
C13	0.6154 (6)	0.1550 (5)	0.5092 (4)	0.0359 (10)	
C14	0.7742 (6)	0.1170 (5)	0.5217 (5)	0.0493 (14)	
H14	0.8203	0.0640	0.5842	0.059*	
C15	0.8573 (6)	0.1570 (5)	0.4444 (5)	0.0478 (13)	
H15	0.9607	0.1315	0.4538	0.057*	
C16	0.7909 (5)	0.2370 (5)	0.3490 (5)	0.0412 (11)	
H16	0.8500	0.2646	0.2960	0.049*	
C17	0.6381 (5)	0.2743 (4)	0.3346 (4)	0.0332 (9)	
C18	0.5478 (5)	0.2331 (4)	0.4165 (4)	0.0318 (9)	
C19	0.6444 (7)	0.3885 (6)	0.1579 (4)	0.0481 (13)	
H19A	0.6959	0.4395	0.1727	0.072*	
H19B	0.5787	0.4328	0.0980	0.072*	
H19C	0.7157	0.3192	0.1447	0.072*	
C20	0.3366 (11)	−0.0190 (7)	0.0837 (7)	0.080 (2)	
H20A	0.4065	0.0260	0.0620	0.119*	
H20B	0.3899	−0.1032	0.1118	0.119*	
H20C	0.2755	0.0094	0.1357	0.119*	
C21	0.2387 (9)	−0.0020 (7)	−0.0081 (6)	0.0687 (19)	
C22	0.1964 (15)	−0.1131 (9)	−0.0076 (9)	0.104 (4)	
H22A	0.1282	−0.1280	0.0464	0.156*	
H22B	0.2846	−0.1817	0.0044	0.156*	
H22C	0.1497	−0.1001	−0.0733	0.156*	

### Atomic displacement parameters (Å^2^)

**Table:**

	*U*^11^	*U*^22^	*U*^33^	*U*^12^	*U*^13^	*U*^23^
Lu1	0.03318 (16)	0.03674 (17)	0.02788 (16)	−0.00642 (11)	−0.00080 (10)	0.00064 (11)
Cu1	0.0320 (3)	0.0311 (3)	0.0231 (3)	−0.0067 (2)	−0.0002 (2)	0.0016 (2)
O1	0.0325 (16)	0.0387 (18)	0.0285 (16)	−0.0012 (14)	−0.0070 (13)	0.0040 (14)
O2	0.0273 (15)	0.0370 (17)	0.0294 (16)	−0.0032 (13)	0.0015 (12)	0.0019 (14)
O3	0.0262 (15)	0.051 (2)	0.0274 (16)	−0.0050 (14)	−0.0056 (13)	0.0061 (15)
O4	0.0310 (17)	0.046 (2)	0.0342 (18)	−0.0116 (15)	0.0022 (14)	0.0008 (16)
O5	0.047 (2)	0.047 (2)	0.041 (2)	−0.0047 (16)	−0.0034 (16)	−0.0095 (17)
O6	0.055 (2)	0.0403 (19)	0.0288 (17)	−0.0128 (16)	0.0039 (15)	−0.0030 (15)
O7	0.070 (3)	0.080 (3)	0.043 (2)	−0.017 (2)	0.007 (2)	−0.026 (2)
O8	0.0387 (19)	0.044 (2)	0.053 (2)	−0.0106 (16)	−0.0028 (17)	−0.0020 (18)
O9	0.063 (3)	0.038 (2)	0.049 (2)	−0.015 (2)	0.004 (2)	−0.0001 (19)
O10	0.067 (3)	0.060 (3)	0.096 (4)	−0.038 (2)	0.022 (3)	−0.030 (3)
O11	0.054 (2)	0.044 (2)	0.038 (2)	−0.0193 (18)	−0.0003 (17)	0.0013 (17)
O12	0.056 (2)	0.046 (2)	0.0371 (19)	−0.0160 (18)	0.0024 (17)	−0.0075 (17)
O13	0.089 (4)	0.044 (2)	0.074 (3)	−0.025 (2)	−0.012 (3)	−0.010 (2)
O14	0.153 (7)	0.058 (3)	0.064 (4)	−0.007 (4)	0.004 (4)	0.006 (3)
N1	0.038 (2)	0.040 (2)	0.0283 (19)	−0.0179 (17)	0.0022 (16)	−0.0051 (17)
N2	0.053 (3)	0.031 (2)	0.028 (2)	0.0008 (18)	0.0008 (18)	0.0038 (17)
N3	0.037 (2)	0.051 (3)	0.036 (2)	−0.0170 (19)	0.0068 (17)	−0.013 (2)
N4	0.043 (2)	0.040 (2)	0.057 (3)	−0.0144 (19)	0.015 (2)	−0.014 (2)
N5	0.043 (2)	0.040 (2)	0.048 (3)	−0.0103 (19)	−0.006 (2)	−0.008 (2)
C1	0.051 (3)	0.047 (3)	0.030 (3)	0.005 (2)	−0.013 (2)	0.005 (2)
C2	0.029 (2)	0.031 (2)	0.032 (2)	−0.0041 (17)	−0.0074 (17)	−0.0076 (19)
C3	0.031 (2)	0.036 (2)	0.045 (3)	−0.0006 (18)	−0.012 (2)	−0.011 (2)
C4	0.026 (2)	0.057 (3)	0.060 (3)	−0.002 (2)	−0.004 (2)	−0.028 (3)
C5	0.028 (2)	0.055 (3)	0.049 (3)	−0.011 (2)	0.005 (2)	−0.022 (3)
C6	0.027 (2)	0.034 (2)	0.041 (2)	−0.0099 (17)	−0.0015 (18)	−0.015 (2)
C7	0.031 (2)	0.027 (2)	0.031 (2)	−0.0072 (16)	−0.0049 (17)	−0.0050 (18)
C8	0.038 (2)	0.042 (2)	0.034 (2)	−0.019 (2)	0.0086 (19)	−0.012 (2)
C9	0.052 (3)	0.056 (3)	0.031 (3)	−0.025 (3)	0.008 (2)	0.004 (2)
C10	0.105 (6)	0.039 (3)	0.038 (3)	−0.032 (3)	0.014 (3)	−0.001 (3)
C11	0.069 (4)	0.069 (4)	0.037 (3)	0.007 (4)	0.010 (3)	0.026 (3)
C12	0.050 (3)	0.035 (2)	0.029 (2)	0.008 (2)	−0.010 (2)	0.000 (2)
C13	0.033 (2)	0.033 (2)	0.034 (2)	0.0005 (18)	−0.0136 (19)	−0.006 (2)
C14	0.040 (3)	0.045 (3)	0.056 (3)	0.003 (2)	−0.025 (3)	−0.016 (3)
C15	0.029 (2)	0.045 (3)	0.066 (4)	−0.004 (2)	−0.015 (2)	−0.017 (3)
C16	0.027 (2)	0.042 (3)	0.056 (3)	−0.0091 (19)	−0.001 (2)	−0.017 (2)
C17	0.028 (2)	0.033 (2)	0.038 (2)	−0.0078 (17)	−0.0056 (18)	−0.0078 (19)
C18	0.029 (2)	0.032 (2)	0.032 (2)	−0.0054 (17)	−0.0025 (17)	−0.0089 (18)
C19	0.043 (3)	0.063 (3)	0.040 (3)	−0.027 (3)	0.011 (2)	−0.006 (3)
C20	0.109 (7)	0.059 (4)	0.069 (5)	−0.026 (4)	0.013 (5)	−0.018 (4)
C21	0.075 (5)	0.062 (4)	0.060 (4)	−0.017 (4)	0.024 (4)	−0.013 (4)
C22	0.125 (9)	0.072 (6)	0.098 (8)	−0.023 (6)	−0.042 (7)	0.000 (5)

### Geometric parameters (Å, °)

**Table:**

Lu1—O3	2.328 (3)	C2—C7	1.428 (6)
Lu1—O2	2.335 (3)	C3—C4	1.386 (8)
Lu1—O6	2.438 (4)	C3—H3	0.9300
Lu1—O12	2.463 (4)	C4—C5	1.365 (8)
Lu1—O1	2.476 (3)	C4—H4	0.9300
Lu1—O11	2.483 (4)	C5—C6	1.432 (7)
Lu1—O8	2.484 (4)	C5—H5	0.9300
Lu1—O5	2.486 (4)	C6—C7	1.365 (7)
Lu1—O4	2.500 (3)	C6—C8	1.449 (7)
Lu1—O9	2.579 (4)	C8—H8	0.9300
Lu1—N5	2.888 (5)	C9—C10	1.496 (9)
Lu1—N3	2.890 (5)	C9—H9A	0.9700
Cu1—O2	1.933 (3)	C9—H9B	0.9700
Cu1—O3	1.943 (4)	C10—C11	1.488 (12)
Cu1—N1	1.968 (4)	C10—H10A	0.9700
Cu1—N2	1.975 (4)	C10—H10B	0.9700
O1—C2	1.380 (6)	C11—H11A	0.9700
O1—C1	1.456 (6)	C11—H11B	0.9700
O2—C7	1.337 (5)	C12—C13	1.451 (8)
O3—C18	1.330 (5)	C12—H12	0.9300
O4—C17	1.376 (6)	C13—C18	1.376 (7)
O4—C19	1.433 (6)	C13—C14	1.427 (7)
O5—N3	1.274 (6)	C14—C15	1.337 (9)
O6—N3	1.262 (6)	C14—H14	0.9300
O7—N3	1.218 (6)	C15—C16	1.406 (8)
O8—N4	1.266 (6)	C15—H15	0.9300
O9—N4	1.266 (7)	C16—C17	1.376 (6)
O10—N4	1.207 (6)	C16—H16	0.9300
O11—N5	1.263 (7)	C17—C18	1.425 (7)
O12—N5	1.262 (6)	C19—H19A	0.9600
O13—N5	1.225 (6)	C19—H19B	0.9600
O14—C21	1.172 (9)	C19—H19C	0.9600
N1—C8	1.285 (7)	C20—C21	1.510 (12)
N1—C9	1.508 (6)	C20—H20A	0.9600
N2—C12	1.283 (7)	C20—H20B	0.9600
N2—C11	1.506 (7)	C20—H20C	0.9600
C1—H1A	0.9600	C21—C22	1.516 (13)
C1—H1B	0.9600	C22—H22A	0.9600
C1—H1C	0.9600	C22—H22B	0.9600
C2—C3	1.383 (6)	C22—H22C	0.9600
			
O3—Lu1—O2	64.14 (12)	O7—N3—O5	122.3 (5)
O3—Lu1—O6	146.60 (13)	O6—N3—O5	115.5 (4)
O2—Lu1—O6	146.96 (12)	O7—N3—Lu1	176.3 (4)
O3—Lu1—O12	73.77 (14)	O6—N3—Lu1	56.7 (2)
O2—Lu1—O12	73.01 (13)	O5—N3—Lu1	59.0 (3)
O6—Lu1—O12	118.90 (13)	O10—N4—O9	122.3 (5)
O3—Lu1—O1	127.07 (12)	O10—N4—O8	121.8 (5)
O2—Lu1—O1	65.77 (11)	O9—N4—O8	115.9 (4)
O6—Lu1—O1	86.29 (13)	O13—N5—O12	120.6 (5)
O12—Lu1—O1	76.49 (13)	O13—N5—O11	122.6 (5)
O3—Lu1—O11	117.79 (14)	O12—N5—O11	116.8 (4)
O2—Lu1—O11	115.27 (14)	O13—N5—Lu1	176.7 (4)
O6—Lu1—O11	67.38 (14)	O12—N5—Lu1	58.0 (3)
O12—Lu1—O11	51.54 (14)	O11—N5—Lu1	58.9 (3)
O1—Lu1—O11	70.71 (13)	O1—C1—H1A	109.5
O3—Lu1—O8	116.27 (13)	O1—C1—H1B	109.5
O2—Lu1—O8	80.52 (13)	H1A—C1—H1B	109.5
O6—Lu1—O8	73.24 (14)	O1—C1—H1C	109.5
O12—Lu1—O8	143.62 (14)	H1A—C1—H1C	109.5
O1—Lu1—O8	70.02 (13)	H1B—C1—H1C	109.5
O11—Lu1—O8	125.09 (13)	O1—C2—C3	125.7 (4)
O3—Lu1—O5	98.38 (13)	O1—C2—C7	114.2 (4)
O2—Lu1—O5	137.85 (13)	C3—C2—C7	120.1 (5)
O6—Lu1—O5	51.62 (13)	C2—C3—C4	119.9 (5)
O12—Lu1—O5	141.90 (13)	C2—C3—H3	120.0
O1—Lu1—O5	130.80 (12)	C4—C3—H3	120.0
O11—Lu1—O5	106.84 (15)	C5—C4—C3	121.0 (5)
O8—Lu1—O5	73.70 (14)	C5—C4—H4	119.5
O3—Lu1—O4	65.10 (12)	C3—C4—H4	119.5
O2—Lu1—O4	125.11 (12)	C4—C5—C6	119.7 (5)
O6—Lu1—O4	87.74 (13)	C4—C5—H5	120.2
O12—Lu1—O4	74.18 (14)	C6—C5—H5	120.2
O1—Lu1—O4	142.40 (12)	C7—C6—C5	120.1 (5)
O11—Lu1—O4	72.73 (14)	C7—C6—C8	122.8 (4)
O8—Lu1—O4	142.20 (14)	C5—C6—C8	116.9 (5)
O5—Lu1—O4	68.93 (13)	O2—C7—C6	123.2 (4)
O3—Lu1—O9	68.24 (15)	O2—C7—C2	117.6 (4)
O2—Lu1—O9	70.71 (14)	C6—C7—C2	119.2 (4)
O6—Lu1—O9	105.25 (15)	N1—C8—C6	127.5 (5)
O12—Lu1—O9	135.82 (14)	N1—C8—H8	116.3
O1—Lu1—O9	109.59 (14)	C6—C8—H8	116.3
O11—Lu1—O9	172.64 (14)	C10—C9—N1	111.6 (5)
O8—Lu1—O9	50.10 (14)	C10—C9—H9A	109.3
O5—Lu1—O9	67.15 (15)	N1—C9—H9A	109.3
O4—Lu1—O9	107.80 (15)	C10—C9—H9B	109.3
O3—Lu1—N5	96.29 (14)	N1—C9—H9B	109.3
O2—Lu1—N5	93.85 (14)	H9A—C9—H9B	108.0
O6—Lu1—N5	93.20 (14)	C11—C10—C9	112.5 (6)
O12—Lu1—N5	25.74 (13)	C11—C10—H10A	109.1
O1—Lu1—N5	71.03 (13)	C9—C10—H10A	109.1
O11—Lu1—N5	25.82 (14)	C11—C10—H10B	109.1
O8—Lu1—N5	139.36 (13)	C9—C10—H10B	109.1
O5—Lu1—N5	127.25 (14)	H10A—C10—H10B	107.8
O4—Lu1—N5	72.30 (13)	C10—C11—N2	112.3 (5)
O9—Lu1—N5	161.55 (16)	C10—C11—H11A	109.1
O3—Lu1—N3	123.65 (13)	N2—C11—H11A	109.1
O2—Lu1—N3	149.96 (12)	C10—C11—H11B	109.1
O6—Lu1—N3	25.65 (12)	N2—C11—H11B	109.1
O12—Lu1—N3	136.02 (13)	H11A—C11—H11B	107.9
O1—Lu1—N3	108.20 (12)	N2—C12—C13	127.2 (4)
O11—Lu1—N3	87.73 (14)	N2—C12—H12	116.4
O8—Lu1—N3	70.14 (13)	C13—C12—H12	116.4
O5—Lu1—N3	26.05 (12)	C18—C13—C14	119.5 (5)
O4—Lu1—N3	78.60 (13)	C18—C13—C12	122.7 (4)
O9—Lu1—N3	85.21 (14)	C14—C13—C12	117.7 (5)
N5—Lu1—N3	112.46 (14)	C15—C14—C13	120.5 (5)
O2—Cu1—O3	79.40 (14)	C15—C14—H14	119.7
O2—Cu1—N1	90.93 (16)	C13—C14—H14	119.7
O3—Cu1—N1	170.32 (15)	C14—C15—C16	121.1 (5)
O2—Cu1—N2	169.55 (17)	C14—C15—H15	119.5
O3—Cu1—N2	90.81 (17)	C16—C15—H15	119.5
N1—Cu1—N2	98.87 (18)	C17—C16—C15	119.6 (5)
O2—Cu1—Lu1	40.28 (10)	C17—C16—H16	120.2
O3—Cu1—Lu1	40.17 (9)	C15—C16—H16	120.2
N1—Cu1—Lu1	130.35 (12)	C16—C17—O4	125.6 (5)
N2—Cu1—Lu1	129.39 (14)	C16—C17—C18	120.0 (5)
C2—O1—C1	117.0 (4)	O4—C17—C18	114.4 (4)
C2—O1—Lu1	118.3 (2)	O3—C18—C13	123.5 (4)
C1—O1—Lu1	122.5 (3)	O3—C18—C17	117.2 (4)
C7—O2—Cu1	129.1 (3)	C13—C18—C17	119.3 (4)
C7—O2—Lu1	123.5 (3)	O4—C19—H19A	109.5
Cu1—O2—Lu1	107.35 (14)	O4—C19—H19B	109.5
C18—O3—Cu1	128.2 (3)	H19A—C19—H19B	109.5
C18—O3—Lu1	124.5 (3)	O4—C19—H19C	109.5
Cu1—O3—Lu1	107.26 (14)	H19A—C19—H19C	109.5
C17—O4—C19	116.9 (4)	H19B—C19—H19C	109.5
C17—O4—Lu1	117.9 (3)	C21—C20—H20A	109.5
C19—O4—Lu1	124.3 (3)	C21—C20—H20B	109.5
N3—O5—Lu1	95.0 (3)	H20A—C20—H20B	109.5
N3—O6—Lu1	97.6 (3)	C21—C20—H20C	109.5
N4—O8—Lu1	99.2 (3)	H20A—C20—H20C	109.5
N4—O9—Lu1	94.7 (3)	H20B—C20—H20C	109.5
N5—O11—Lu1	95.3 (3)	O14—C21—C20	123.0 (8)
N5—O12—Lu1	96.3 (3)	O14—C21—C22	122.6 (9)
C8—N1—C9	114.8 (4)	C20—C21—C22	114.4 (7)
C8—N1—Cu1	125.0 (3)	C21—C22—H22A	109.5
C9—N1—Cu1	120.1 (3)	C21—C22—H22B	109.5
C12—N2—C11	114.2 (5)	H22A—C22—H22B	109.5
C12—N2—Cu1	124.9 (4)	C21—C22—H22C	109.5
C11—N2—Cu1	120.8 (4)	H22A—C22—H22C	109.5
O7—N3—O6	122.2 (5)	H22B—C22—H22C	109.5
			
O3—Lu1—Cu1—O2	163.0 (2)	O3—Lu1—O8—N4	−20.1 (4)
O6—Lu1—Cu1—O2	−98.9 (4)	O2—Lu1—O8—N4	−75.0 (3)
O12—Lu1—Cu1—O2	80.80 (19)	O6—Lu1—O8—N4	125.3 (3)
O1—Lu1—Cu1—O2	6.99 (19)	O12—Lu1—O8—N4	−118.4 (3)
O11—Lu1—Cu1—O2	78.7 (2)	O1—Lu1—O8—N4	−142.5 (3)
O8—Lu1—Cu1—O2	−63.48 (19)	O11—Lu1—O8—N4	170.7 (3)
O5—Lu1—Cu1—O2	−137.7 (2)	O5—Lu1—O8—N4	71.3 (3)
O4—Lu1—Cu1—O2	152.41 (19)	O4—Lu1—O8—N4	62.4 (4)
O9—Lu1—Cu1—O2	−101.1 (2)	O9—Lu1—O8—N4	−2.3 (3)
N5—Lu1—Cu1—O2	79.12 (19)	N5—Lu1—O8—N4	−159.8 (3)
N3—Lu1—Cu1—O2	−127.1 (2)	N3—Lu1—O8—N4	98.5 (3)
O2—Lu1—Cu1—O3	−163.0 (2)	O3—Lu1—O9—N4	165.0 (4)
O6—Lu1—Cu1—O3	98.1 (4)	O2—Lu1—O9—N4	96.0 (3)
O12—Lu1—Cu1—O3	−82.2 (2)	O6—Lu1—O9—N4	−49.6 (4)
O1—Lu1—Cu1—O3	−156.0 (2)	O12—Lu1—O9—N4	132.5 (3)
O11—Lu1—Cu1—O3	−84.3 (2)	O1—Lu1—O9—N4	41.9 (4)
O8—Lu1—Cu1—O3	133.5 (2)	O8—Lu1—O9—N4	2.3 (3)
O5—Lu1—Cu1—O3	59.3 (2)	O5—Lu1—O9—N4	−85.2 (3)
O4—Lu1—Cu1—O3	−10.6 (2)	O4—Lu1—O9—N4	−142.2 (3)
O9—Lu1—Cu1—O3	95.9 (2)	N5—Lu1—O9—N4	130.5 (4)
N5—Lu1—Cu1—O3	−83.9 (2)	N3—Lu1—O9—N4	−65.8 (3)
N3—Lu1—Cu1—O3	69.9 (2)	O3—Lu1—O11—N5	−36.4 (4)
O3—Lu1—Cu1—N1	177.2 (2)	O2—Lu1—O11—N5	36.4 (3)
O2—Lu1—Cu1—N1	14.2 (2)	O6—Lu1—O11—N5	−179.8 (3)
O6—Lu1—Cu1—N1	−84.7 (4)	O12—Lu1—O11—N5	−1.7 (3)
O12—Lu1—Cu1—N1	94.99 (19)	O1—Lu1—O11—N5	86.1 (3)
O1—Lu1—Cu1—N1	21.19 (18)	O8—Lu1—O11—N5	132.6 (3)
O11—Lu1—Cu1—N1	92.9 (2)	O5—Lu1—O11—N5	−145.7 (3)
O8—Lu1—Cu1—N1	−49.28 (19)	O4—Lu1—O11—N5	−84.9 (3)
O5—Lu1—Cu1—N1	−123.50 (19)	N3—Lu1—O11—N5	−163.7 (3)
O4—Lu1—Cu1—N1	166.61 (18)	O3—Lu1—O12—N5	150.0 (3)
O9—Lu1—Cu1—N1	−86.9 (2)	O2—Lu1—O12—N5	−142.7 (3)
N5—Lu1—Cu1—N1	93.32 (19)	O6—Lu1—O12—N5	3.7 (4)
N3—Lu1—Cu1—N1	−112.9 (2)	O1—Lu1—O12—N5	−74.3 (3)
O3—Lu1—Cu1—N2	−19.2 (3)	O11—Lu1—O12—N5	1.7 (3)
O2—Lu1—Cu1—N2	177.8 (2)	O8—Lu1—O12—N5	−97.5 (4)
O6—Lu1—Cu1—N2	78.9 (4)	O5—Lu1—O12—N5	67.2 (4)
O12—Lu1—Cu1—N2	−101.4 (2)	O4—Lu1—O12—N5	82.0 (3)
O1—Lu1—Cu1—N2	−175.2 (2)	O9—Lu1—O12—N5	−178.6 (3)
O11—Lu1—Cu1—N2	−103.5 (2)	N3—Lu1—O12—N5	28.1 (4)
O8—Lu1—Cu1—N2	114.3 (2)	O2—Cu1—N1—C8	−9.7 (4)
O5—Lu1—Cu1—N2	40.1 (2)	N2—Cu1—N1—C8	174.0 (4)
O4—Lu1—Cu1—N2	−29.8 (2)	Lu1—Cu1—N1—C8	−18.8 (5)
O9—Lu1—Cu1—N2	76.7 (2)	O2—Cu1—N1—C9	170.2 (4)
N5—Lu1—Cu1—N2	−103.1 (2)	N2—Cu1—N1—C9	−6.2 (4)
N3—Lu1—Cu1—N2	50.7 (2)	Lu1—Cu1—N1—C9	161.1 (3)
O3—Lu1—O1—C2	−26.9 (4)	O2—Cu1—N2—C12	33.1 (13)
O2—Lu1—O1—C2	−6.9 (3)	O3—Cu1—N2—C12	12.8 (5)
O6—Lu1—O1—C2	155.0 (3)	N1—Cu1—N2—C12	−167.5 (5)
O12—Lu1—O1—C2	−84.1 (3)	Lu1—Cu1—N2—C12	25.1 (6)
O11—Lu1—O1—C2	−137.7 (3)	O2—Cu1—N2—C11	−150.3 (9)
O8—Lu1—O1—C2	81.5 (3)	O3—Cu1—N2—C11	−170.6 (6)
O5—Lu1—O1—C2	126.4 (3)	N1—Cu1—N2—C11	9.0 (6)
O4—Lu1—O1—C2	−123.6 (3)	Lu1—Cu1—N2—C11	−158.4 (5)
O9—Lu1—O1—C2	50.1 (3)	Lu1—O6—N3—O7	175.8 (5)
N5—Lu1—O1—C2	−110.3 (3)	Lu1—O6—N3—O5	−5.6 (4)
N3—Lu1—O1—C2	141.5 (3)	Lu1—O5—N3—O7	−175.9 (5)
O3—Lu1—O1—C1	170.5 (4)	Lu1—O5—N3—O6	5.5 (4)
O2—Lu1—O1—C1	−169.5 (4)	O3—Lu1—N3—O6	−158.5 (3)
O6—Lu1—O1—C1	−7.6 (4)	O2—Lu1—N3—O6	105.5 (3)
O12—Lu1—O1—C1	113.3 (4)	O12—Lu1—N3—O6	−56.6 (3)
O11—Lu1—O1—C1	59.7 (4)	O1—Lu1—N3—O6	32.6 (3)
O8—Lu1—O1—C1	−81.1 (4)	O11—Lu1—N3—O6	−36.2 (3)
O5—Lu1—O1—C1	−36.2 (4)	O8—Lu1—N3—O6	92.5 (3)
O4—Lu1—O1—C1	73.8 (4)	O5—Lu1—N3—O6	−174.1 (5)
O9—Lu1—O1—C1	−112.5 (4)	O4—Lu1—N3—O6	−109.1 (3)
N5—Lu1—O1—C1	87.0 (4)	O9—Lu1—N3—O6	141.7 (3)
N3—Lu1—O1—C1	−21.2 (4)	N5—Lu1—N3—O6	−43.8 (3)
O3—Cu1—O2—C7	−167.2 (4)	O3—Lu1—N3—O5	15.5 (3)
N1—Cu1—O2—C7	12.5 (4)	O2—Lu1—N3—O5	−80.5 (4)
N2—Cu1—O2—C7	172.2 (9)	O6—Lu1—N3—O5	174.1 (5)
Lu1—Cu1—O2—C7	−178.2 (5)	O12—Lu1—N3—O5	117.4 (3)
O3—Cu1—O2—Lu1	11.07 (16)	O1—Lu1—N3—O5	−153.3 (3)
N1—Cu1—O2—Lu1	−169.23 (17)	O11—Lu1—N3—O5	137.8 (3)
N2—Cu1—O2—Lu1	−9.6 (11)	O8—Lu1—N3—O5	−93.4 (3)
O3—Lu1—O2—C7	168.3 (4)	O4—Lu1—N3—O5	65.0 (3)
O6—Lu1—O2—C7	−28.7 (5)	O9—Lu1—N3—O5	−44.3 (3)
O12—Lu1—O2—C7	88.5 (3)	N5—Lu1—N3—O5	130.2 (3)
O1—Lu1—O2—C7	6.0 (3)	Lu1—O9—N4—O10	178.5 (5)
O11—Lu1—O2—C7	58.2 (4)	Lu1—O9—N4—O8	−3.8 (5)
O8—Lu1—O2—C7	−66.3 (3)	Lu1—O8—N4—O10	−178.3 (5)
O5—Lu1—O2—C7	−118.8 (3)	Lu1—O8—N4—O9	3.9 (5)
O4—Lu1—O2—C7	144.2 (3)	Lu1—O12—N5—O13	176.5 (5)
O9—Lu1—O2—C7	−117.2 (4)	Lu1—O12—N5—O11	−2.9 (5)
N5—Lu1—O2—C7	73.2 (3)	Lu1—O11—N5—O13	−176.6 (5)
N3—Lu1—O2—C7	−78.6 (4)	Lu1—O11—N5—O12	2.9 (5)
O3—Lu1—O2—Cu1	−10.08 (14)	O3—Lu1—N5—O12	−28.8 (3)
O6—Lu1—O2—Cu1	152.90 (17)	O2—Lu1—N5—O12	35.5 (3)
O12—Lu1—O2—Cu1	−89.90 (17)	O6—Lu1—N5—O12	−176.8 (3)
O1—Lu1—O2—Cu1	−172.4 (2)	O1—Lu1—N5—O12	98.3 (3)
O11—Lu1—O2—Cu1	−120.20 (17)	O11—Lu1—N5—O12	−177.0 (5)
O8—Lu1—O2—Cu1	115.35 (17)	O8—Lu1—N5—O12	115.5 (3)
O5—Lu1—O2—Cu1	62.8 (2)	O5—Lu1—N5—O12	−134.4 (3)
O4—Lu1—O2—Cu1	−34.2 (2)	O4—Lu1—N5—O12	−90.2 (3)
O9—Lu1—O2—Cu1	64.44 (18)	O9—Lu1—N5—O12	3.1 (6)
N5—Lu1—O2—Cu1	−105.20 (16)	N3—Lu1—N5—O12	−159.3 (3)
N3—Lu1—O2—Cu1	103.0 (2)	O3—Lu1—N5—O11	148.1 (3)
N2—Cu1—O3—C18	−18.0 (4)	O2—Lu1—N5—O11	−147.5 (3)
Lu1—Cu1—O3—C18	176.8 (5)	O6—Lu1—N5—O11	0.2 (3)
O2—Cu1—O3—Lu1	−11.09 (16)	O12—Lu1—N5—O11	177.0 (5)
N2—Cu1—O3—Lu1	165.2 (2)	O1—Lu1—N5—O11	−84.8 (3)
O2—Lu1—O3—C18	−166.9 (4)	O8—Lu1—N5—O11	−67.6 (4)
O6—Lu1—O3—C18	29.9 (5)	O5—Lu1—N5—O11	42.6 (4)
O12—Lu1—O3—C18	−88.3 (4)	O4—Lu1—N5—O11	86.8 (3)
O1—Lu1—O3—C18	−146.6 (3)	O9—Lu1—N5—O11	−179.9 (4)
O11—Lu1—O3—C18	−60.6 (4)	N3—Lu1—N5—O11	17.7 (3)
O8—Lu1—O3—C18	129.4 (4)	C1—O1—C2—C3	−9.0 (7)
O5—Lu1—O3—C18	53.5 (4)	Lu1—O1—C2—C3	−172.6 (4)
O4—Lu1—O3—C18	−8.5 (4)	C1—O1—C2—C7	170.9 (4)
O9—Lu1—O3—C18	114.7 (4)	Lu1—O1—C2—C7	7.3 (5)
N5—Lu1—O3—C18	−75.7 (4)	O1—C2—C3—C4	−179.4 (5)
N3—Lu1—O3—C18	46.7 (4)	C7—C2—C3—C4	0.7 (7)
O2—Lu1—O3—Cu1	10.02 (14)	C2—C3—C4—C5	0.3 (8)
O6—Lu1—O3—Cu1	−153.12 (18)	C3—C4—C5—C6	−1.2 (8)
O12—Lu1—O3—Cu1	88.66 (19)	C4—C5—C6—C7	1.1 (7)
O1—Lu1—O3—Cu1	30.3 (2)	C4—C5—C6—C8	−174.4 (5)
O11—Lu1—O3—Cu1	116.33 (18)	Cu1—O2—C7—C6	−8.1 (6)
O8—Lu1—O3—Cu1	−53.7 (2)	Lu1—O2—C7—C6	173.9 (3)
O5—Lu1—O3—Cu1	−129.56 (18)	Cu1—O2—C7—C2	173.4 (3)
O4—Lu1—O3—Cu1	168.4 (2)	Lu1—O2—C7—C2	−4.6 (5)
O9—Lu1—O3—Cu1	−68.34 (19)	C5—C6—C7—O2	−178.6 (4)
N5—Lu1—O3—Cu1	101.26 (19)	C8—C6—C7—O2	−3.3 (7)
N3—Lu1—O3—Cu1	−136.39 (16)	C5—C6—C7—C2	−0.1 (7)
O3—Lu1—O4—C17	8.1 (3)	C8—C6—C7—C2	175.1 (4)
O2—Lu1—O4—C17	32.0 (4)	O1—C2—C7—O2	−2.1 (6)
O6—Lu1—O4—C17	−151.9 (3)	C3—C2—C7—O2	177.8 (4)
O12—Lu1—O4—C17	87.2 (3)	O1—C2—C7—C6	179.3 (4)
O1—Lu1—O4—C17	127.2 (3)	C3—C2—C7—C6	−0.8 (7)
O11—Lu1—O4—C17	141.1 (4)	C9—N1—C8—C6	−176.8 (5)
O8—Lu1—O4—C17	−93.3 (4)	Cu1—N1—C8—C6	3.1 (7)
O5—Lu1—O4—C17	−102.5 (4)	C7—C6—C8—N1	5.9 (8)
O9—Lu1—O4—C17	−46.6 (4)	C5—C6—C8—N1	−178.7 (5)
N5—Lu1—O4—C17	114.0 (4)	C8—N1—C9—C10	149.4 (5)
N3—Lu1—O4—C17	−127.7 (4)	Cu1—N1—C9—C10	−30.5 (7)
O3—Lu1—O4—C19	176.4 (4)	N1—C9—C10—C11	75.7 (7)
O2—Lu1—O4—C19	−159.7 (4)	C9—C10—C11—N2	−72.4 (8)
O6—Lu1—O4—C19	16.4 (4)	C12—N2—C11—C10	−158.0 (6)
O12—Lu1—O4—C19	−104.5 (4)	Cu1—N2—C11—C10	25.1 (9)
O1—Lu1—O4—C19	−64.5 (5)	C11—N2—C12—C13	179.4 (6)
O11—Lu1—O4—C19	−50.6 (4)	Cu1—N2—C12—C13	−3.8 (9)
O8—Lu1—O4—C19	74.9 (5)	N2—C12—C13—C18	−6.9 (9)
O5—Lu1—O4—C19	65.8 (4)	N2—C12—C13—C14	176.5 (6)
O9—Lu1—O4—C19	121.7 (4)	C18—C13—C14—C15	0.3 (8)
N5—Lu1—O4—C19	−77.7 (4)	C12—C13—C14—C15	177.0 (5)
N3—Lu1—O4—C19	40.6 (4)	C13—C14—C15—C16	−0.1 (9)
O3—Lu1—O5—N3	−167.0 (3)	C14—C15—C16—C17	−0.5 (8)
O2—Lu1—O5—N3	132.6 (3)	C15—C16—C17—O4	−178.4 (5)
O6—Lu1—O5—N3	−3.3 (3)	C15—C16—C17—C18	0.9 (7)
O12—Lu1—O5—N3	−92.6 (3)	C19—O4—C17—C16	2.8 (7)
O1—Lu1—O5—N3	34.3 (3)	Lu1—O4—C17—C16	171.9 (4)
O11—Lu1—O5—N3	−44.5 (3)	C19—O4—C17—C18	−176.6 (4)
O8—Lu1—O5—N3	78.0 (3)	Lu1—O4—C17—C18	−7.4 (5)
O4—Lu1—O5—N3	−107.8 (3)	Cu1—O3—C18—C13	13.5 (7)
O9—Lu1—O5—N3	131.0 (3)	Lu1—O3—C18—C13	−170.2 (4)
N5—Lu1—O5—N3	−62.4 (3)	Cu1—O3—C18—C17	−168.2 (3)
O3—Lu1—O6—N3	33.6 (4)	Lu1—O3—C18—C17	8.1 (6)
O2—Lu1—O6—N3	−117.8 (3)	C14—C13—C18—O3	178.5 (5)
O12—Lu1—O6—N3	138.5 (3)	C12—C13—C18—O3	1.9 (8)
O1—Lu1—O6—N3	−149.1 (3)	C14—C13—C18—C17	0.2 (7)
O11—Lu1—O6—N3	140.2 (3)	C12—C13—C18—C17	−176.4 (5)
O8—Lu1—O6—N3	−78.9 (3)	C16—C17—C18—O3	−179.1 (4)
O5—Lu1—O6—N3	3.3 (3)	O4—C17—C18—O3	0.2 (6)
O4—Lu1—O6—N3	68.0 (3)	C16—C17—C18—C13	−0.7 (7)
O9—Lu1—O6—N3	−39.8 (3)	O4—C17—C18—C13	178.6 (4)
N5—Lu1—O6—N3	140.1 (3)		
